# Retraction: Low expression of miR-30a-5p induced the proliferation and invasion of oral cancer via promoting the expression of FAP

**DOI:** 10.1042/BSR-2017-1027_RET

**Published:** 2024-11-15

**Keywords:** FAP, miR-30a-5p, Oral cancer

This article “Low expression of miR-30a-5p induced the proliferation and invasion of oral cancer via promoting the expression of FAP” DOI: 10.1042/BSR20171027 is being retracted from *Bioscience Reports* at the request of the Editor-in-Chief and the Editorial Board. This follows receipt of a notification from a reader, alerting the Editorial Board to:

- Signs of digital splicing in Figure 1F GAPDH Western blot between SCC-15 and SCC-25 protein bands
- Figure 3B Tca-8113 inhibitor panel seems to appear as Figure 1G pcDNA LINC00511 panel from Ding et al, 2018 (DOI: 10.1111/jop.12677)
- Figure 3B SCC-15 NC panel which seems to appear as Figure 3B SCC-15 FAP panel with rotation, and as Figure 3C Mock panel from Ding et al, 2018 (DOI: 10.1111/jop.12677) with rotation
- Figure 4A SCC-15 24h mimics panel which also seems to appear as Figure 4A SCC-15 24h siFAP panel
- Figure 4B SCC-15 siFAP panel seems to also appear as Figure 4B SCC-15 mimics+siFAP panel

The Editorial Office has identified additional concerns regarding similarities between Figure 2E Tca-8113 FAP mimics and siFAP protein bands, as well as potential signs of digital splicing in Figure 2E Tca-8113 panel. The authors were contacted regarding the concerns raised, and stated the original data could not be located. Given the extent of the issues raised, the Editorial Board stand by the decision to retract the article. The authors agree to the retraction.

